# Occurrence, Definition and Risk Factors Related to Groin Wound Complications Following Open Vascular Surgeries

**DOI:** 10.1111/iwj.70843

**Published:** 2026-01-29

**Authors:** Andreas L. H. Gerken, Yuting Jiang, Christel Weiß, Lillian Schmoll, Johannes Eberhard, Christoph Reißfelder, Martin Sigl, Klaus Amendt, Kay Schwenke

**Affiliations:** ^1^ Division of Vascular Surgery, Department of Surgery University Medical Center Mannheim, Medical Faculty Mannheim, Heidelberg University Mannheim Germany; ^2^ Department of Surgery, University Medical Center Mannheim, Medical Faculty Mannheim Heidelberg University Mannheim Germany; ^3^ Department of Medical Statistics and Biomathematics, University Medical Center Mannheim, Medical Faculty Mannheim Heidelberg University Mannheim Germany; ^4^ Division of Angiology, Interdisciplinary Vascular Center University Medical Center Mannheim, Medical Faculty Mannheim, Heidelberg University Mannheim Germany

**Keywords:** endarterectomy, lymphocele, postoperative complications, surgical wound infection, treatment outcome

## Abstract

Open femoral vessel access is commonly performed in vascular surgery, but surgical site complications (SSCs) occur frequently. The aim of this study is to evaluate the incidence and identify potential risk factors by applying a new standardised definition and grading of various types of groin wound complications. This retrospective analysis includes 201 consecutive patients with 219 vertical groin incisions to expose the femoral vessels for different vascular interventions. A prophylactic drain was placed intraoperatively in almost all incisions (91%). Groin SSCs were defined and graded into four categories according to a modified Clavien‐Dindo classification. Potential risk factors were evaluated using univariable analysis. For multivariable analysis, a multiple logistic regression was performed. Cutoff values were determined through ROC analysis. According to the proposed definition, regular postoperative course grade 0 (no SSC) occurred in 163 patients (74.4%), grade 1 (minor SSC) in 10 (4.6%), grade 2 (moderate SSC) in 14 (6.4%), and grade 3/4 (major or life‐threatening SSC) in 32 (14.6%) incisions. The incidence of clinically relevant SSCs (grade 2–4) was 21%. Drainage volume was an independent parameter that predicted relevant SSCs with a threshold value of 70 mL/24 h on postoperative day 4 (sensitivity 100%; specificity 67%; AUC = 0.835; *p* = 0.0004). Groin wound complications following vascular procedures are common. Lymphatic leakage appears to be the most significant, potentially preventable condition associated with relevant SSCs. Prophylactic or early therapeutic interventions should focus on reducing lymphatic morbidity.

## Key Points

This single‐center retrospective cohort study analyzed 219 vertical groin incisions for vascular access regarding the occurrence of wound complications. These are the key findings:

- Relevant groin wound complications after vascular procedures occurred in 21% of cases.
- Lymphatic leakage was associated significantly to high incidence rates.
- Drainage volume on postoperative day 4 predicted complicated wound scenarios.
- The management of wounds and drainage should be optimized.
- A standardized definition facilitates the evaluation of prophylactic interventions.

## 1 Introduction

Groin incisions provide access to the femoral vessels for treating occlusions, aneurysms, or controlling bleeding. Due to demographic changes, the prevalence of peripheral artery disease (PAD) and the demand for revascularization procedures are increasing globally [1, 2, 3]. More than 200 million people worldwide are affected by PAD [1]. Despite advances in endovascular treatment, international guidelines on PAD still recommend open surgical endarterectomy as the gold standard for treating lesions involving the common femoral artery due to its higher long‐term patency [4, 5, 6]. According to the AHA's annual report, a total number of 75 710 embolectomies/endarterectomies and 56 705 peripheral arterial bypasses were performed in 2021 in the United States alone [1].

However, the groin region is a common site for surgical site complications (SSCs). The aetiology and types of SSCs are heterogeneous. They may occur intraoperatively or in the postoperative period and include infections, vascular injury, and damage to lymphatic tissue, which may lead to lymphatic leakage and the formation of lymphoceles. The definition of postoperative infectious complications (surgical site infections, SSIs) by the Centers for Disease Control and Prevention (CDC) is widely used and accepted [7]. In contrast, studies defining noninfectious and lymphatic SSCs are scarce, as research involving vascular patients primarily focuses on revascularization outcomes. Despite advances in prevention and surgical technique, SSIs remain a significant cause of postoperative morbidity. The commonly reported incidence of SSIs after vascular procedures in the groin in the literature ranges between 8% and 17% [8, 9, 10, 11]. Still, rates up to 31% have been published [12]. Pooled SSI rates after arterial interventions have been published in a systematic review [13]. The overall SSI incidence was 8.1%. The reported incidence differed depending on the vascular pathology (occlusive vs. aneurysmal), the SSI definition used, and the trial design. The authors detected a higher incidence in the control arms of RCTs (16%), as opposed to an SSI rate of 7.2% in observational studies. Potential reasons for an increased infection incidence in the groin compared to incisions outside the groin might be the bacterial burden in the groin, the higher density of lymphatic vessels, comorbidities like diabetes or obesity, and the impairment of microperfusion in vascular patients. The morbidity and economic burden of postoperative SSIs are well known. SSIs lead to an increase in health care expenditure [14], prolong the hospital stay, reduce the quality of life [15], and contribute to a higher mortality rate [9, 10, 16]. Therefore, measures to reduce the occurrence of SSIs are essential.

We hypothesised that lymphatic leakage, a potentially modifiable complication, contributes to the increased incidence of SSCs in the groin region. The aim of this study was to evaluate a standardised definition of SSCs following vascular procedures involving the femoral vessels to determine the incidence of clinically relevant groin wound complications and to identify factors associated with SSCs while considering postoperative drainage characteristics.

## 2 Methods

### 2.1 Study Design and Procedure

A retrospective monocentric cohort study of patients who had an open vascular access in the groin for various types of vascular procedures between January 2021 and December 2022 at the Division of Vascular Surgery at a University Hospital was performed. Patients were identified consecutively based on an internal database. The study adhered to the STROBE guidelines [17] and was conducted in accordance with the Declaration of Helsinki.

### 2.2 Data Acquisition

Data were extracted from electronic patient records and paper‐based records, including patient demographics, comorbidities, procedure characteristics, postoperative outcomes, such as the daily and cumulative discharge of intraoperatively inserted suction drains and their postoperative management, as well as 90‐day surgical site complications (SSCs), 90‐day mortality rate, need for reintervention or reoperation, and length of hospital stay. Depending on availability, records of the primary stay and subsequent outpatient controls were considered.

### 2.3 Surgical Procedures and Postoperative Management

A perioperative antibiotic prophylaxis with cefuroxime was administered 30–60 min before surgery. Five different specialist surgeons performed the vascular procedures. Vascular access to the femoral artery or vein was standardised. We used a vertical lateral approach to medialize and spare the lymphatic tissue to avoid injuring lymphatic vessels. Before arterial clamping, patients received 100 IU of heparin per kg. After endarterectomy, we exclusively used bovine patches (XenoSure Biologic Patch, LeMaitre Vascular GmbH, Sulzbach, Germany). For aortofemoral bypasses, we used prosthetic material (Intergard Synergy Knitted, Getinge GmbH, Rastatt, Germany). For femoropopliteal bypasses, an autologous vein was preferred. If this was not available, prosthetic grafts were used instead (GORE PROPATEN Vascular Graft, W.L. Gore & Associates GmbH, Putzbrunn, Germany). The wound was closed with an absorbable fascial and subcutaneous suture and an absorbable intracutaneous skin suture. Standard dressings were used to cover the wound. No patient received prophylactic sNPWT (single‐use closed incision negative pressure wound therapy). According to our centre's common practice, a suction drainage (12 Ch. Redon drainage with PRIVAC high‐vacuum systems, Primed Halberstadt Medizintechnik GmbH, Halberstadt, Germany) was inserted adjacent to the vessel before closing the fascial level. Postoperative drainage management was not standardised during the study period. Usually, drainage was removed if the wound secretion was less than 50 mL per day. Conservative treatment included the administration of i.v. antibiotics in the case of an SSI, wound therapy, immobilisation, and local compression. Postoperative interventions (e.g., aspiration, percutaneous or prolonged drainage, lymphography, sclerotherapy, radiation) or operations to treat SSCs were indicated at the discretion of the treating physicians.

### 2.4 Outcomes and Definitions

The primary outcome was SSC within 90 days of surgery. The different types of SSCs were defined according to Table 1. The severity grading of SSCs was performed retrospectively based on a standardised classification: As displayed in detail in Table 2, the classification contains both noninfectious SSCs (defined according to Clavien‐Dindo [23]) and infectious SSCs (defined according to CDC [7], or Szilagyi [22] in case of vascular graft involvement, respectively). The severity was divided into four categories (minor, moderate, major, major life threatening/‐limiting SSCs) depending on the management strategy (conservative, interventional, surgical, ICU). Each groin incision was categorised into one specific grade of SSC according to the most severe grade. If a bilateral approach was utilised, both sites were evaluated separately regarding the occurrence of SSCs.

**TABLE 1:** Different types and definitions of common surgical site complications (SSCs) in the groin.

Type	Definition	Adapted from
Noninfectious SSCs		
− bleeding; synonymous haemorrhage, including anastomotic/erosion bleeding	(Painful) swelling of the wound with perivascular colour flow in duplex sonography, or contrast extravasation on CT angiography, or relevant drop in haemoglobin level Persistent discharge of blood from the surgically inserted drains, or from the wound	
− hematoma	Localised collection of blood outside the blood vessels, within the tissue or the wound cavity	Healy, 2016 [18]
− seroma	Localised collection of serous fluid within the tissue or the wound cavity	Healy, 2016 [18]
− wound dehiscence/breakdown	Rupture of the wound suture line	Healy, 2016 [18]
− wound necrosis	Occurrence of nonviable tissue at the wound ground or wound edges	
− Lymphatic complications		
lymphatic leakage; synonymous lymphatic drainage, lymphatic fistula	Persistent discharge of lymphatic fluid (≥ 50 mL/24 h, > 5 postoperative days) from the surgically inserted drains or from the wound in the absence of a drainage	Gerken, 2020 [19]; Uhl, 2020 [20]; Twine, 2013 [21]
Lymphorrhea; synonymous lymphocutaneous fistula, wound drainage	Persistent leakage of lymphatic fluid (≥ 50 mL/24 h, > 5 postoperative days) from the wound requiring more than one dressing change per day due to dressing material sodden with lymphatic fluid	Gerken, 2020 [19]; Uhl, 2020 [20]; Twine, 2013 [21]
Lymphocele; synonymous lymphocyst	Postoperative accumulation of clear fluid in the wound cavity lacking epithelial lining in the absence of wound dehiscence, hematoma, or infection.	Gerken, 2020 [19]; Twine, 2013 [21]
Infectious SSCs		
− surgical site infection (SSI) including: cellulitis, erysipelas, abscess, vascular graft infection	CDC definitions and diagnostic criteria for superficial, deep, and organ/space SSI Szilagyi classification for prosthetic vascular graft infection: − Grade I: involvement of the skin (cellulitis/dermal infection); − Grade II: involvement of the subcutaneous tissue; − Grade III: involvement of the vascular prosthesis	Official definition of CDC by NHS^a^; Szilagyi, 1972 [22]

^a^ National Healthcare Safety Network, Centers for Disease Control and Prevention (CDC). Surgical Site Infection (SSI) Event. 2017. Available at: https://www.cdc.gov/nhsn/pdfs/pscmanual/9pscssicurrent.pdf, accessed October 14, 2025.

**TABLE 2:** Incidence of wound complications following vascular procedures in the groin classified according to the proposed definition and severity grading of SSCs (*n* = 219).

Grade	Short title	Management	Incidence	Description
0	No SSC	None	163 (74.4%)	Primary postoperative wound healing
1	Minor SSC	Conservative	10 (4.6%)	*Minor SSCs, conservative management:* Wound complications leading to deviation from the normal postoperative course which can be managed *conservatively* (e.g., compression therapy, immobilisation, prolonged drainage > 50 mL/24 h > 5 days). CD grades I, and II with conservative management.
2	Moderate SSC	Interventional	14 (6.4%)	*Moderate SSCs, interventional management:* Wound complication (e.g., symptomatic lymphocele, hematoma, minor wound dehiscence, superficial SSI according to CDC, Szilagyi I) leading to complicated/prolonged postoperative course or readmission that need and require *intervention* (e.g., aspiration, drainage, sclerotherapy, lymphography, radiotherapy, intensive local wound care, iv antibiotics). CD grades II with readmission for i.v. antibiotics/wound care, and grade III with reintervention.
3	Major SSC		18 (8.2%)	*Major SSCs, surgical management:* Any type of major wound complication (e.g., hematoma, bleeding, pain, necrosis, wound breakdown, chronic lymphocele, lymphorrhea, deep SSI according to CDC, Szilagyi II) requiring *reoperation* (e.g., surgical wound revision, vacuum therapy). CD grade III with reoperation.
4	Major life‐threatening/−limiting SSC	Surgical/ICU	14 (6.4%)	*Life‐threatening SSC:* Wound complication with impact on patients' life (e.g., vascular graft infection, organ space SSI according to CDC, Szilagyi III, anastomotic/erosion bleeding, sepsis, death) usually requiring *ICU and/or surgical* management. CD grades IV, and V.

*Note:* Postoperative wound complications grades 2–4 were regarded as clinically relevant SSCs (grey background).

Abbreviations: CD, Clavien‐Dindo; ICU, Intensive Care Unit; SSC, surgical site complication; SSI, surgical site infection defined according to Centers for Disease Control and Prevention (CDC).

### 2.5 Statistical Analysis

Statistical analysis was conducted using SAS statistical software, release 9.4 (SAS Institute Inc., Cary, NC, USA). For qualitative data, absolute and relative frequencies were estimated. For quantitative data that are approximately normally distributed, mean values and standard deviations were calculated. For ordinal‐scaled or skewed variables, the median value and interquartile range (IQR) were considered instead.

For each postoperative day, a univariable logistic regression analysis was performed to evaluate associations between a binary outcome (i.e., clinically relevant SSC) and drainage volume on that day as an independent variable. The area under the ROC curve (AUC) quantified the predictive power of the relevant statistical model to identify the most appropriate day for predicting the outcome. Odds ratios (OR) were assessed, along with 95% confidence intervals. Furthermore, we estimated the optimal cutoff at which the Youden index (sensitivity+specificity−1) is maximal.

To compare two groups in univariable models concerning a quantitative normally distributed factor, the two‐sample *t*‐test was applied. Otherwise, the Wilcoxon two‐sample test was used as a location test. For categorical factors, the Chi‐square test was applied. Only if the preconditions of the Chi‐square test were not fulfilled (under the null hypothesis, expected frequencies less than 5), Fisher's exact test was used instead. For ordinally scaled variables, the Cochran‐Armitage trend test was performed.

All variables with *p* values < 0.2 were eligible for inclusion in multivariable models for clinically relevant SSCs (grades 2–4). Multivariable logistic regression was utilised to calculate odds ratios and 95% confidence intervals (CIs) for 90‐day SSCs using the SAS ‘Selection = Backward’ option. For this analysis, the significance level was set at < 0.10 in order to be able to detect more potential risk factors. The results of all other calculations were considered statistically significant if a *p*‐value < 0.05 was obtained.

## 3 Results

### 3.1 Demographics

The study population consisted of 201 patients with 219 groin incisions for vascular access (i.e., 18 patients had a bilateral approach). Patient and procedure characteristics are presented in Table 3. The mean age of the included patients was 67 years, and 58% were male. Regarding co‐morbidities, 28.9% of the patients had concomitant diabetes mellitus (type II in 96.6%), and 90% of the patients were classified as ASA III or IV, indicating severe systemic disease. More than half of the patients (61.5%) were active smokers or had a history of smoking. Most patients underwent arterial revascularization for advanced stages of PAD, i.e., intermittent claudication (IC) with failure of conservative treatment, or critical limb‐threatening ischemia (CLTI). Nearly half of the patients (42%) underwent endarterectomy of the femoral artery, followed by bypass surgery in 17%. The proportion of venous procedures was only 2%, and prosthetic graft material was used in 15% of the procedures. A small proportion of patients (16%) had an open or infected pedal wound before surgery.

**TABLE 3:** Patient characteristics and interventions (*n* = 201).

Patient characteristics	
Sex (♀:♂)	84 (42%): 117 (58%)
Age (years)	67 ± 13
BMI (kg/m^2^)	26.98 ± 6.43
Comorbidities and risk factors	
Obesity^a^	42 (21%)
Diabetes mellitus	58 (29%)
Coronary artery disease	102 (51%)
COPD	40 (20%)
Arterial hypertension	152 (76%)
CKD	64 (32%)
PAD	146 (73%)
Fontaine Stage II (IC)	40 (20%)
Fontaine Stage III‐IV (CLTI)	103 (51%)
Current smoker	97 (49%)
Recent smoker	26 (13%)
Anticoagulants	65 (33%)
ASA class	
II	19 (9.6%)
III	133 (67.5%)
IV	45 (22.8%)
Procedures	
Femoral endarterectomy	84 (42%)
Femoral to popliteal bypass	34 (17%)
Surgical repair of femoral pseudoaneurysm	30 (15%)
Surgical decanulation of VA‐ECMO	22 (11%)
Arterial thrombectomy	13 (6%)
Aorto‐bifemoral bypass	8 (4%)
Other^b^	10 (5%)
Procedure characteristics	
Prosthetic bypass graft	31 (15%)
Re‐do operation	38 (19%)
Inpatient details	
Duration of postoperative hospital stay (days)	9 (5–19)
Drainage details	
Duration of drainage (days)	2 (1–4)
≤ 2 days	136 (68%)
≤ 4 days	62 (31%)
≤ 10 days	9 (4.5%)
> 10 days	9 (4.5%)

*Note:* The data is presented as absolute and relative values (percentages), mean ± standard deviation, or median together with interquartile range (IQR).

Abbreviations: ASA, American Society of Anesthesiologists; BMI, body mass index; CKD, chronic kidney disease; CLTI, chronic limb‐threatening ischemia; COPD, chronic obstructive pulmonary disease; IC, intermittent claudication; PAD, peripheral arterial occlusive disease; VA‐ECMO, venoarterial extracorporal membrane oxygenation.

^a^ Obesity defined as BMI ≥ 30 kg/m^2^.

^b^ Other procedures: venous incision or thrombectomy (*n* = 4), arteriovenous fistula repair (*n* = 3), cutdown technique for endovascular iliac stenting (*n* = 2), arterialization (*n* = 1).

### 3.2 Incidence, Types, and Severity of SSCs

Of the 219 groin incisions analysed, 163 (74.4%) healed without any wound complications. In 10 (4.6%) incisions, a minor surgical site complication (SSC) was observed, which could be treated conservatively. In 42 (21.0%) cases, a clinically relevant SSC requiring interventional or surgical treatment (grades 2–4) occurred within 90 days after the vascular procedure. The incidence of various types of SSCs is presented in Table 4, and the severity grading of SSCs, classified according to the proposed definition, is presented in Table 2. We performed 30 reoperations due to different types of SSCs (13.7%). Among these, 18 groin wounds were classified as grade 3, requiring surgical wound revision, while 12 groin wounds were classified as grade 4 due to bypass infection (*n* = 5), erosion bleeding (*n* = 2), or death associated with groin wounds (*n* = 5). In 4 cases a sartorius muscle flap was used for vascular coverage. The overall in‐hospital mortality rate was 13.7%, and the 90‐day mortality rate was 19.6%. The median postoperative duration of hospital stay was 9 days (IQR: 5–19). The median lengths of hospital stay was significantly longer in patients with SSCs classified as grades 2–4 compared to those with grades 0–1 (18 vs. 8 days, IQR: 7–30 vs. 5–16 days; *p* = 0.0002).

**TABLE 4:** Documented surgical site complications (SSCs) during the postoperative course after vascular procedures in the groin (*n* = 219).

SSC type	Frequency
Lymphatic	
Lymphatic leakage	30 (13.7%)
Lymphocele	24 (11.0%)
Infectious	
Wound infection	25 (11.4%)
Graft infection Szilagy III	8 (3.7%)
Erosion bleeding	4 (1.8%)
Noninfectious	
Wound healing disorder	19 (8.7%)
Hematoma	16 (7.3%)
Wound dehiscence	11 (5.0%)

*Note:* Overlapping SSCs possible.

### 3.3 Postoperative Drainage and Risk Factor Analysis

**FIGURE 1:**
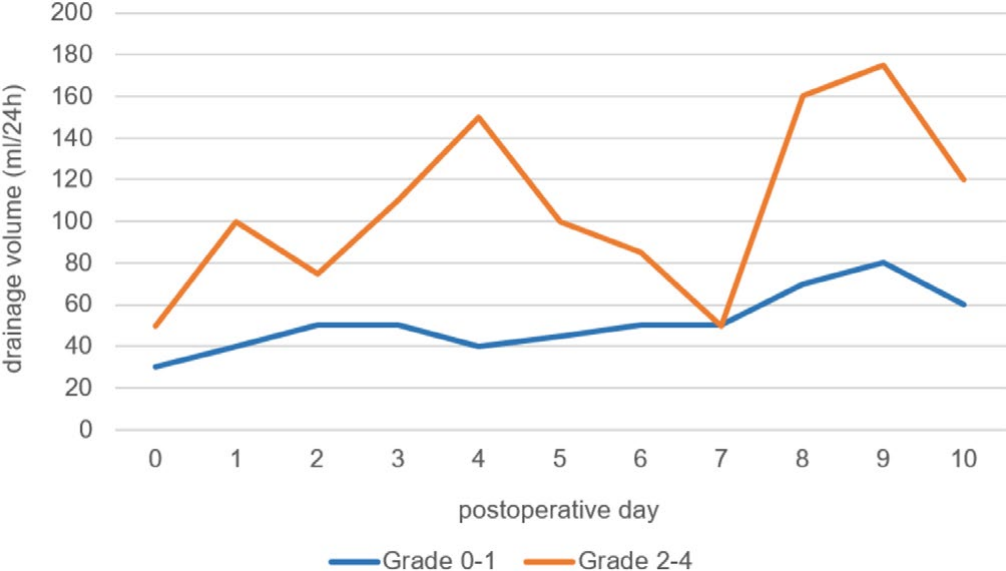
Course of median daily drainage volumes on postoperative days 1–10 in patients with and without clinically relevant surgical site complications shows a significant difference on postoperative day 4 (*p* = 0.0004). Sample sizes for days 0 to 10: Grade 0–1 (blue line): *N* = 158, 120, 73, 49, 35, 29, 18, 13, 9, 6, 5; grade 2–4 (orange line): *N* = 41, 33, 26, 22, 13, 12, 11, 6, 5, 4, 4.

Suction drains were placed in 199 (91%) of 219 incisions, i.e., in only 20 incisions no drain was inserted intraoperatively. The median duration of drainage was 2 days (IQR: 1–4). Suction drains were removed at a median drainage output of 22.5 (IQR: 10–50) ml/24 h. Daily postoperative drainage volumes were higher in groin incisions with clinically relevant SSCs (grades 2–4). A significant difference was observed on postoperative day 4 (40 vs. 150 mL/24 h, *p* = 0.0004). The cutoff value for drainage volume on postoperative day 4, calculated by ROC analysis for predicting a clinically relevant SSC, was 70 mL/24 h (*p* = 0.0320; AUC = 0.835; OR = 1.824 per 100 mL; sensitivity 100%; specificity 67%, *n* = 48). The course of median daily postoperative drainage volumes for both groups is presented in Figure 1. No significant difference was found in daily drainage volumes on days 0–3. However, early cumulative drainage volumes for postoperative days 0–2, 0–3, and 0–4 differed significantly between the groups (*p* = 0.0299, *p* = 0.0127, *p* = 0.0115, respectively). Aside from drainage volume on postoperative day 4, univariable analysis identified cumulative drainage volume until drainage removal (*p* = 0.0116), coronary artery disease (*p* = 0.0137), arterial hypertension (*p* = 0.0381), and bypass surgery (*p* = 0.0149) as factors associated with clinically relevant SSCs (Table 5). However, the only significant parameter remaining when trying to create a multivariable model was cumulative drainage volume (*p* = 0.0024, OR 1.090 for a 100 mL increase in drainage volume). Hence, cumulative drainage volume differed significantly between the subgroups (with and without complication) with medians of 180 [60; 890] ml and 90 [30; 230] ml, respectively (*n* = 199). We found an optimal cutoff value of 371 mL (*p* = 0.0024, AUC = 0.628; OR = 1.090 per 100 mL) connected with a reasonable specificity of 84% but a rather low value for sensitivity (41%). Hence, the drainage volume on day 4 is of greater prognostic value. As it is difficult to accurately measure cumulative drainage volume in everyday clinical practice, a second multivariable analysis was conducted, excluding cumulative drainage volume. In this model, only drainage volume on postoperative day 4 was associated with SSCs grades 2–4 (*p* = 0.0379).

**TABLE 5:** Uni‐ and multivariable assessment of factors associated with clinically relevant surgical site complications (SSC grade 2–4).

Univariable analysis				Multvariable analysis*		
Characteristic	SSC grade 0–1 *n* = 173 (79%)	SSC grade 2–4 *n* = 46 (21%)	*p*‐value	OR	95% CI	*p*‐value
Age (years)	66.3 ± 12.9	67.9 ± 13.1	0.4384			
Gender (♂:♀)	98 (56.7%): 75 (43.3%)	33 (71.7%): 13 (28.3%)	0.0635			0.4093
Diabetes	44 (25.6%)	16 (34.8%)	0.2146			0.4468
COPD	32 (18.6%)	11 (23.9%)	0.4216			
Coronary artery disease	77 (44.8%)	30 (65.2%)	**0.0137**			0.7375
Hypertension	124 (72.1%)	40 (87.0%)	**0.0381**			0.5354
PAD			0.2339			
CKD	55 (32.0%)	13 (28.3%)	0.6289			
Smoking	83 (48.3%)	26 (56.5%)	0.3691			
Obesity	36 (20.9%)	9 (20.0%)	0.8910			
BMI (kg/m^2^)	26.84 (±6.52)	27.27 (±5.55)	0.6779			
ASA class			0.9507			
II	17 (10.1%)	3 (6.5%)				
III	112 (66.3%)	34 (73.9%)				
IV	40 (23.7%)	9 (19.6%)				
Oral anticoagulation	48 (28.2%)	18 (40.0%)	0.1281			0.5413
Re‐do operation	35 (20.2%)	11 (23.9%)	0.5858			
Femoral to popliteal bypass	23 (13.3%)	13 (28.3%)	**0.0149**			
Graft material:			0.2491			
− none	83 (48.0%)	16 (34.8%)				
− natural	61 (35.3%)	19 (41.3%)				
− prosthetic	29 (16.8%)	11 (23.9%)				
Pedal wound	25 (14.5%)	11 (23.9%)	0.1771			
Drainage volume at removal (mL/24 h)	20 (0–950)	30 (0–270)	0.1795			
Drainage volume on POD 4 (mL/24 h)	40 (0–640)	150 (70–400)	0.0004	1.771	1.032–3.039	**0.0379** ***
Cumulative drainage volume (mL)	90 (0–2550)	180 (0–3260)	0.0116	1.090	1.031–1.153	**0.0024** **

*Note:* *P*‐values in univariable analysis for ASA‐class and graft material globally refer to different subclasses. Therefore there are bold entries in the subclasses. OR and 95% CI in multivariable analysis are only provided for significant factors (drainage volume on POD 4 and cumulative drainage volume).

* 0.1 significance level.

** *n* = 199.

*** *n* = 48; POD, postoperative day.

## 4 Discussion

An open approach to the femoral vessels is necessary for various vascular procedures. In this study, wound complications following vascular procedures in the groin were systematically defined, and their incidence and potential risk factors were determined. Clinically relevant surgical site complications (SSCs) occurred in 21%, confirming the postoperative morbidity associated with groin incisions. Postoperative drainage volume was associated with a complicated wound course. Patients experiencing relevant SSCs faced significantly longer postoperative hospital stays (8 vs. 18 days), negatively impacting treatment costs. The high incidence of adverse events emphasises the need for effective preventive and early therapeutic measures.

The Groin Wound Infection after Vascular Exposure (GIVE) prospective cohort study identified female sex, obesity, ischemic heart disease, aqueous betadine skin preparation, bypass/patch use, and increased operative time as independent predictors of SSIs [10]. A large retrospective trial utilising American registry data further revealed COPD, dialysis, and preoperative hyponatremia [9]. A recently published retrospective cohort study, including patients who underwent vascular procedures in the groin, found a SSC rate of 39.1% in obese patients compared to 14.1% in non‐obese patients [24]. Potential risk factors in our univariable analysis include coronary artery disease, hypertension, and bypass surgery. Most of the identified parameters are not modifiable and represent the morbidity of the included population.

Furthermore, previous studies have overlooked drainage volume as a potential risk factor. The results of the present study identified cumulative drainage volume as the only independent factor significantly associated with clinically relevant SSCs (grades 2–4) in the multivariable analysis. Excluding cumulative drainage volume, the isolated drainage volume on postoperative day 4 emerged as the only significant factor associated with clinically relevant SSCs (OR 1.8). The reported incidence of lymphatic complications after vascular procedures ranges from 0.5% to 11.5% [20, 25, 26, 27, 28, 29]. Compared to our results, lymphatic morbidity appears to be underestimated in the literature. This is most likely due to detection bias arising from the retrospective design of previously published studies evaluating lymphatic morbidity in the groin. However, lymphatic morbidity may be indirectly reflected in the high incidence rates of SSIs following groin incisions reported in previous studies, reaching up to 30% [12] compared to incisions outside the groin. Although our study is also retrospective, we have nevertheless focused on extracting the most comprehensive data possible according to the criteria mentioned. Concerning wound infections, our results show an overall SSI rate of 11.4%. Deep infections (Szilagy III) occurred in 3.7%. These rates are comparable to pooled SSI rates from the literature [13].

Regarding the reporting and incidence of SSCs, it is irrational to consider different types of SSCs because there is considerable overlap, and management strategies are similar. Postoperative lymphatic leakage due to the unintended injury to lymphatic tissue in the groin may present clinically as persistent drainage from surgically inserted drains or lead to serous discharge through the incision, causing secondary wound breakdown and bacterial superinfection [21]. In the latter case, it would be counted as an SSI. In addition to the definition of postoperative complications by Clavien‐Dindo [23], we therefore suggest a standardised definition and severity grading of SSCs in the groin, disregarding pathogenesis and concentrating on the invasiveness of the required management strategy. The proposed severity grading (presented in Table 2) is an adaptation of the classification by Clavien‐Dindo. Still, it includes different types of noninfectious and infectious SSCs, particularly lymphatic morbidity. It reflects the implications of SSCs in the groin, specifically in four dimensions: Firstly, on symptoms and patients' lives, secondly, on invasiveness of management, thirdly, on required surgical and medical expertise, fourthly, on health care resources.

In contrast to Clavien‐Dindo, a patient without a groin wound complication returning to the ICU for cardiac or pulmonary reasons would be classified as grade 0. Furthermore, the proposed definition distinguishes between a nonsurgical and a surgical intervention, but does not differentiate between the types of anaesthesia. Similar definitions have been published for lymphatic complications after kidney transplantation [30], and inguinal lymph node dissection [19]. Interestingly, the same threshold value predicting a complicated wound course has been identified in a different cohort of patients after soft tissue tumour resection on the lower limb [31]. Therefore, 70 mL/24 h seems to be the amount of lymphatic secretion that exceeds the resorptive capacity of extraabdominal artificial wound cavities.

**FIGURE 2:**
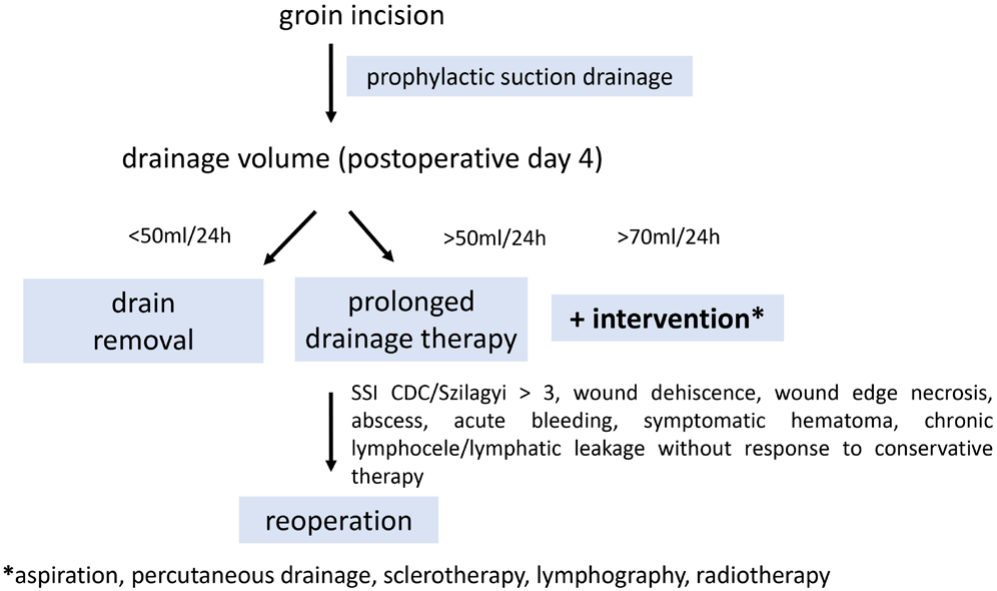
Standardised wound and drainage management after open vascular access in the groin.

Wound and drainage care after groin incisions should be optimised, as evidence‐based guidelines are currently lacking. Current ESVS/SVS guidelines on the management of CLTI, for instance, neither comment on the preferred direction of incision (vertical vs. transverse) nor on indications for intraoperative drain placement [32]. They further lack criteria for safe drainage removal, postoperative incision management, and groin wound care, while WHO global guidelines for the prevention of SSIs [33], NICE medical technologies guidance [34], and ECRI clinical evidence assessment [35] recommend the postoperative use of single‐use closed incision negative pressure wound therapy (sNPWT) to reduce the risk of SSIs in high‐risk wounds. Based on the findings of this study, we summarise our recommendations for postoperative wound management in Figure 2. We recommend considering consistent intraoperative prophylactic drain placement with the intention to possibly reduce SSCs and indicate the postoperative wound course. According to our results, on postoperative day 5, the drainage volume from the fourth postoperative day allows for individual risk stratification and early detection of potentially complicated wound situations. We recommend drain removal when the output rate is less than 50 mL/24 h; otherwise, drainage therapy should be continued. If the drainage volume exceeds 70 mL/24 h, a minimally invasive intervention should be considered to prevent grade 3/4 complications. Success rates as high as 96.8% have been reported for lymphography with lipiodol, depending on drainage volume before the intervention [36]. However, heterogeneous sites of lymphatic leakage have been included. The therapeutic effect of sclerotherapy by instillation of doxycycline or other sclerosing agents was evaluated in smaller case series [37, 38, 39]. Low‐dose radiotherapy has been proposed as an effective therapy for postoperative lymphatic leakage [40, 41]. In future trials, the proposed definition could be employed to compare the therapeutic effect of different interventions to treat lymphatic leakage. We recommend considering surgical wound revision and debridement with or without the use of negative pressure wound therapy (NPWT) in cases of wound dehiscence, wound edge necrosis, superficial abscess, symptomatic hematoma, chronic lymphocele, or lymphatic leakage without response to conservative therapy. In Szilagyi III graft infection [22] or erosion bleeding, the ESVS guidelines on managing vascular graft infection recommend a reoperation with removal of the infected prosthetic graft and reconstruction with autologous material [42]. This can also be combined with muscle flaps and/or NPWT.

The effect of prophylactic measures to reduce SSIs, including the direction of incision, use of prophylactic flaps, adhesive drapes, and topical antibiotics, remains unclear, according to a systematic review published in 2023 [43]. A multi‐institutional randomised controlled trial found a significantly higher incidence of overall wound complications (including infection, wound breakdown and lymphatic leak) in vertical incision (47.5%) compared to transverse (12.7%) (*p* = 0.0001) [44]. Yet, a Cochrane review published in 2020 found no differences in the incidence of lymphoceles and lower certainty evidence that transverse incision resulted in lower risk of wound infection [45]. In alignment with these conflicting results, we exclusively performed the traditional vertical (longitudinal) incisions in the groin because simultaneous exposure of distal iliac and femoral vessels is facilitated while ensuring a preparation line along the lymphatic channels. Prophylactic sNPWT significantly reduced SSIs in vascular surgery [46, 47, 48] with a RR of 0.55 compared to standard dressings in the subgroup analysis of the largest meta‐analysis [48]. Still, several other meta‐analyses failed to show a significant reduction of noninfectious complications by sNPWT [49, 50, 51]. Prophylactic drain placement seems to be justified in combination with vertical incisions because 13.7% of patients in our study had lymphatic leakage and 11% had lymphocele. Prolonged drainage therapy might prevent secondary complications in these cases. Prophylactic suction drains may promote wound healing by preventing fluid accumulation and lymphocele formation that limit the adherence of the wound surfaces. Notably, the most recent Cochrane review evaluating the effect of suction drains after groin incisions concluded that their benefit could not be assessed due to the limited amount and poor quality of published studies. The authors identified three RCTs only, all with a high risk of bias and heterogeneous endpoint definitions [18]. Currently, there is no clear evidence on whether the use of prophylactic drains is beneficial or harmful. A systematic review evaluating different co‐interventions in arterial surgery concluded that subcuticular sutures and sNPWT are able to reduce SSIs [11]. A standardised definition of SSCs would allow the evaluation of such prophylactic interventions in future studies.

## 5 Limitations

The retrospective design limits the validity of the present study. Incorrect documentation of drainage volumes or wound conditions would lead to information, attrition, or detection bias. A larger and prospective data acquisition would be needed in further trials to minimise this kind of bias. Despite the limited sample size, significant intergroup differences were detected in our uni‐ and multivariable analyses, and ROC analysis provided a good AUC for the prediction of SSCs grades 2–4. While the calculated threshold value for drainage output seems to be an ideal screening parameter, specificity was only 67%. This observation could be explained by the fact that in 33% of cases, other SSCs besides lymphatic complications necessitated reoperation or resulted in patient deterioration. Although it was the most suitable predictor in the present study, the cutoff value has to be interpreted with caution because there was a 78% dropout rate of patients on postoperative day four due to prior drainage removal. Larger prospective trials should be performed to validate the proposed classification system with focus on the identification of earlier predictors. Furthermore, the direction of incision was not subject of the study, since we exclusively performed vertical incisions for the reasons mentioned above. The recommendation to consistently use prophylactic drains cannot directly be supported by our results, because we did not collect data on drain‐related adverse events. However, the determination of drainage volumes and its use for risk stratification would be impossible without inserting a drain. We are aware that it is not common practice (drain use 50% in GIVE vs. 91% here) and drains might also be harmful (causing discomfort, immobilisation, or infection). But, as mentioned above, currently there is no argument against prophylactic drains in the literature owing to the lack of valid studies.

## 6 Conclusions

In this retrospective study, postoperative drainage volume on postoperative day four was associated with clinically relevant wound complications open vascular access in the groin. Since lymphatic leakage seems to contribute significantly to high SSC rates, we propose prophylactic drainage. A standardised definition facilitates the reporting of SSCs in future trials. Further research applying standardised endpoint definitions is necessary to validate cutoff values for drainage volume and assess the benefits of preventive interventions to reduce the incidence of major SSCs (grades 3 and 4) after groin incisions, as these are potentially life‐threatening and commonly require intensive care and/or surgical management.

## Funding

The authors have nothing to report.

## Ethics Statement

This retrospective study has been approved by the responsible ethics committee of the Medical Faculty Mannheim, University of Heidelberg (ID 2022‐846 on 09/29/2022).

## Conflicts of Interest

A.L.H.G. has given presentations for Smith & Nephew concerning sNPWT, unrelated to this publication. The authors declare no conflicts of interest.

## Data Availability

The data that support the findings of this study are available on request from the corresponding author. The data are not publicly available due to privacy or ethical restrictions.
